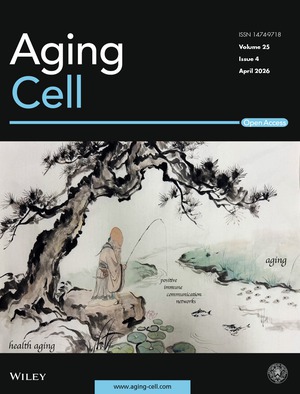# Additional Cover

**DOI:** 10.1111/acel.70522

**Published:** 2026-04-19

**Authors:** Liwei Qiu, Chen Dong, Rui Zhao, Huiyuan Ye, Jian‐lin Gao, Yizhi Chen, Chi Sun, Zhifeng Gu

## Abstract

The cover image is based on the article *Single‐Cell Profiling Reveals Distinct Immune Communication Networks in Centenarians and Elderly Controls* by Liwei Qiu et al., https://doi.org/10.1111/acel.70486.